# Looking through the FOG: microbiome characterization and lipolytic bacteria isolation from a fatberg site

**DOI:** 10.1099/mic.0.001117

**Published:** 2021-12-06

**Authors:** Elizabeth K. Court, Roy R. Chaudhuri, Rahul V. Kapoore, Raffaella X. Villa, Jagroop Pandhal, Catherine A. Biggs, Graham P. Stafford

**Affiliations:** ^1^​ Integrated BioScience Group, School of Clinical Dentistry, University of Sheffield, Sheffield, UK; ^2^​ Department of Civil and Structural Engineering, University of Sheffield, Sheffield, UK; ^3^​ Department of Molecular Biology and Biotechnology, University of Sheffield, Sheffield, UK; ^4^​ Department of Chemical and Biological Engineering, University of Sheffield, Sheffield, UK; ^5^​ Department of Biosciences, College of Science, Swansea University, Swansea, UK; ^6^​ Department of Engineering and Sustainable Development, De Montfort University, Leicester, UK; ^7^​ Environmental Engineering Group, School of Engineering, Newcastle University, Newcastle, UK

**Keywords:** wastewater, fatberg, FOG, fat oil and grease, microbial communities

## Abstract

Sewer systems are complex physical, chemical and microbial ecosystems where fats, oils and grease (FOG) present a major problem for sewer management. Their accumulation can lead to blockages (‘Fatbergs’), sewer overflows and disruption of downstream wastewater treatment. Further advancements of biological FOG treatments need to be tailored to degrade the FOG, and operate successfully within the sewer environment. In this study we developed a pipeline for isolation of lipolytic strains directly from two FOG blockage sites in the UK, and isolated a range of highly lipolytic bacteria. We selected the five most lipolytic strains using Rhodamine B agar plates and pNP-Fatty acid substrates, with two *Serratia* spp., two *Klebsiella* spp. and an environmental *Acinetobacter* strain that all have the capacity to grow on FOG-based carbon sources. Their genome sequences identified the genetic capacity for fatty acid harvesting (lipases), catabolism and utilization (Fad genes). Furthermore, we performed a preliminary molecular characterization of the microbial community at these sites, showing a diverse community of environmental bacteria at each site, but which did include evidence of sequences related to our isolates. This study provides proof of concept to isolation strategies targeting Fatberg sites to yield candidate strains with bioremediation potential for FOG in the wastewater network. Our work sets the foundation for development of novel bioadditions tailored to the environment with non-pathogenic *Acinetobacter* identified as a candidate for this purpose.

## Introduction

Sewer systems are an essential component of society, conveying large amounts of domestic and industrial wastes to treatment facilities for safe disposal in dry weather, and additionally safe and effective management of large volumes of rainwater in wet weather. They are complex physical, chemical and microbiological ecosystems. In this context, the entry of fat, oils and grease (FOG) into the wastewater system from both domestic and food service establishment (FSE) sources has become a major problem for the management of wastewater. Indeed, the UK has seen several high-profile blockages caused by FOG deposits in sewers in recent years, phenomena that have now been termed ‘fatbergs’ [1–4]. Such is the public prominence of these entities, the term fatberg has now entered the English lexicon with inclusion in the Oxford Dictionary in 2015 [5].

The formation of fatbergs is caused by several factors. Firstly, FOGs enter the wastewater system in the form of animal or vegetable fat residues (as triglycerides and free fatty acids), from both domestic, industrial and FSE sources [6–8]. Secondly, these FOGs combine with other material in wastewater systems, such as disposable wipes, to agglomerate forming blockages. In severe cases, the fatbergs can form blockages that are hundreds of metres in length, weigh several metric tonnes and require removal by laborious, dangerous and expensive manual means [9–12]. If left either undetected or untreated, the blockages in sewer networks can lead to sewer overflows, adverse impacts on the environment, for example, through depletion of oxygen in streams, as well as urban flooding [13]. Recent evidence also suggests that this is an increasing problem worldwide – potentially due to rapid population growth and changes in behaviour [12, 14].

While the exact mechanism by which these deposits form is not fully known, a number of factors are thought to contribute. It has been hypothesized that during saponification, free fatty acids (FFAs) combine with calcium and sodium salts in the wastewater to form a nucleation point, leading to solid FOG-soap deposits [8]. It is currently thought that saponification of FFAs, as well as solidification of FOGs in the sewer system, contributes to blockage formation [1, 10, 15]. There is also debate regarding the role of microbes in both the initiation and perpetuation of fatbergs: for example, it has been proposed that bacteria may facilitate the release of calcium salts into the environment – contributing to saponification of FFAs or that, those that release FFA but do not utilize it, can release FFA that causes problems downstream [1, 8, 16]. Another factor is the increased introduction of used cooking oil into wastewater systems, which has a larger amount of FFAs compared to unheated oil and has been shown to be a major cause of the initial nucleation of the FOG [17]. The above factors sit alongside well established culprits of sewer blockages, such as non-flushables like food packaging, condoms and ‘flushable’ wet wipes [12].

One long proposed potential mitigation approach to FOG accumulation is the use of products composed of spore-producing single species or off-the-shelf bacterial consortia that are not adapted to the wide environment. These are deployed in the sewer system to speed up the degradation of blockages. If successful, these products have the potential to save time and money for water companies in terms of money spent clearing fatbergs, which has been estimated at £100 million per year in the UK [18]. However, whilst attractive, the use of lipolytic bacteria that do not utilize FOG, to break down FOGs can lead to the release of FFAs, rather than their consumption or an overall reduction in the sewers. Rather, these FFAs can move downstream from sites of accumulation where they can be subject to saponification and deposit elsewhere [12]. An alternative is the use of more specific fat degrading and consuming bacteria or combinations thereof as part of active FOG degrading microbial consortia that have been considered for Grease Interceptors [19]. Ideally, such a consortium would biodegrade FOG using secreted enzymes, such as lipases, cleaving the ester bond linking FFA with their glycerol backbone breaking down lipids into FFAs, which will then be transported inside the bacterial cells and used as growth substrates. However, the wastewater system is made up of a range of environments, including FSE effluents, sewers, pumping stations and treatment works; all of which have different environmental parameters of temperature, pH, salinity, flow rate, etc. To enable efficient degradation and metabolism of FOG, a consortia with a range of lipases, which target different fatty acids present in FOG, in addition to different rates and abilities to metabolize or assimilate this FOG and that, importantly, are adapted to these different environments would be beneficial.

In the last 20 years, many FOG-degrading products have been tested by water companies in the field and in the laboratory with mixed success, mainly due to a lack of reliability and predictability of the activity of these products in different environments within the wastewater system [1, 19]. This is at least in part due to their inability to thrive or survive in different parts of the system. One reason may be that in many cases these products are single species or microbial consortia isolated from non-sewer environments, i.e. these are largely lab-adapted lipolytic organisms with long shelf lives. In addition, bioadditions have to work in a different way if targeting the deposits. The fatberg and lipid-rich wastewater are two very different substrates and different products need to be created to treat them.

Our aim in this paper was the isolation and screening of bacteria for lipase activity from real fatberg sites, followed by preliminary characterization of their ability to grow in media mimicking wastewater with a FOG-related carbon source. We also characterized, for the first time, the composition of two exemplar fatberg microbial communities, revealing a broad diversity of organisms. We present our data on a selection of lipolytic isolates and propose that this approach will allow development of FOG-degrading consortia tailored to wastewater environments.

## Experimental procedures

### Sampling, bacterial growth and Isolation

Samples were taken from two fatberg sites (site 1 and site 2) in the sewer network in collaboration with a UK water company and contracted personnel. Enrichments were performed using rich growth media (Tryptic Soy Broth) and synthetic wastewater (SWWa). The carbon:nitrogen:phosphorus ratio in the SWWa is 100 : 5 : 1, which translates to a weight ratio of 800 mg l^−1^ COD: 40 mg l^−1^ ammonia-N: 8 mg l^−1^ inorganic phosphorus. This consists of the following per litre: dH_2_O (pH 7): 0.0245 g K_2_HPO_4_; 0.014 g KH_2_PO_4_; 0.16 g NH_4_Cl; 0.6 g MgSO_4_.7H_2_O; 0.07 g CaCl_2_.2H_2_O; 0.01 g EDTA; 2 ml trace mineral. Trace mineral amounts per litre: 1.5 g FeCl_3_.6H_2_O; 0.15 g H_3_BO_3_; 0.03 g CuSO_4_.5H_2_O; 0.03 g KI; 0.12 g MnCL_2_.4H_2_O; 0.06 Na_2_MoI_4_.2H_2_O; 0.12 ZnSO_4_.7H_2_O; 0.15 g CoCl_2_.6H_2_O (Karunakaran E., personal correspondence).

The media contained acetic acid (14 mM) as carbon source with the addition of either olive oil (1 % v/v) or solid fatberg FOG material (1 % w/v), and was incubated with agitation for 3 days at 15–20 °C before being transferred to new flasks of either TSB or SWWa and enriched for up to 5 days at 15–20 °C [20]. These enrichments were then spread onto both TSB or SWWa agar plates that also contained Rhodamine B (0.0001 %) and olive oil (1 %) and screened for lipolytic activity using lipase assays (see below) and taken forward for analysis [21]. Stocks of isolates from this study were stored in glycerol at −80 °C.

Growth studies of strains were carried out in synthetic wastewater with either addition of FOG (1 % olive oil) or FOG plus acetate (14 mM, 0.81 mg ml^−1^). The strains were grown at 25C in a Tecan Sunrise in 96-well plates with horizontal shaking, OD_600_ measurements taken at 30 intervals, and all wells having respective control triplicates containing media, media plus oil/ acetate or oil +acetate, which were subtracted from the culture positive wells to rule out any emulsification or precipitation effects. Growth studies were carried out in at least triplicate with technical triplicates in each run.

### Fatty acid methyl ester profiling

All chemicals and analytical reagents were of high-performance liquid chromatography grade (Sigma-Aldrich, Dorset, UK) unless stated otherwise. Fatberg samples (~5 to 7 mg) were weighed followed by direct transesterification as described elsewhere [22–24]. Briefly, 300 µl of toluene and 300 µl of 0.5M sodium methoxide were added to the weighed fatberg samples, followed by incubation at 80 °C for 20 min. After cooling to room temperature, 300 µl of 10 % boron tri-fluoride in methanol was added and the mixture incubated at 80 °C for 20 min. After cooling to room temperature, 300 µl water and 600 µl of hexane were added. The mixture was vortexed for 1 min and centrifuged at 18 000 ***g*** at 4 °C for 10 min. The organic phase was recovered, measured and evaporated to dryness under inert nitrogen gas. The dried fatty acid methyl esters (FAMEs) were reconstituted in 80 µl hexane prior to identification and quantification as described elsewhere [24]. In total, five technical replicates were run, among which only the FAMEs identified in three or more replicates were considered true hits. The data was later normalized to dry weight of the samples and FAME’s were reported on a percentage basis.

### DNA isolation, 16S rRNA sequencing and bioinformatics

Total DNA extractions from swabs of the sewer wall at the air:liquid interface were resuspended in TE buffer were carried out using DNeasy PowerSoil kit following manufacturer’s instructions (Qiagen). DNA quality was assessed using a nanodrop spectrophotometer before being sent to MR DNA (MR DNA, Shallowater, TX, USA) where 16S rRNA V3/V4 variable regions were amplified using primers (341F: CCTACGGGNGGCWGCAG; 806R: GGACTACHVGGGTWTCTAAT; [25]) with barcodes on the forward primer and MiSeq adapters following manufacturer guidelines. Sequencing data were processed using the MR DNA analysis pipeline where the final OTUs were taxonomically classified using blastn against a curated database derived from RDPII [26] and NCBI [27]. Heatmaps were generated using Morpheus [28] and PCA analysis performed using METAGENassist [29] PERMANOVA and ANOVA statistical analysis were performed with phyloseq., as implemented in the tool MicrobiomeAnalyst [30, 31]. These sequence data have been submitted to the DDBJ/EMBL/GenBank databases under accession number (ERS4556234), while OTU level data and frequencies are present in the Supplementary Material files (available in the online version of this article).

### Genome sequencing

Genomic DNA from isolated lipase producers were carried out using Wizard Genomic DNA Purification Kit (Promega) before sequencing at MicrobesNG, Birmingham. Genomic DNA libraries were prepared using Nextera XT Library Prep Kit (Illumina, San Diego, USA) using Hamilton Microlab STAR automated handling system, following the manufacturer’s protocol with the following modifications: 2 ng DNA was used as input and PCR elongation for 1 min. Pooled libraries were quantified using Kapa Biosystems Library Quantification Kit for Illumina on a Roche light cycler 96 qPCR machine. Libraries were sequenced on the Illumina HiSeq using a 250 bp paired end protocol. Reads were adapter trimmed using Trimmomatic version 0.30 with a sliding window quality cut off of Q15 [32]. *De novo* assembly was performed on samples using SPAdes version 3.7 [33] and contigs were annotated using Prokka [34]. Table 1 indicates accession numbers at the EMBL database.

For 16S-based phylogeny, all available complete genomes of *Serratia *, *Klebsiella* and *Acinetobacter* were downloaded, and the longest 16S rRNA gene from each was identified using Barrnap [35]. Following initial phylogenetic analysis, the strains most closely related to the five SFB genomes were identified. The 16S rRNA sequences were aligned using muscle [36], and redundant sequences and sequences shorter than 90 % of the length of the longest sequence were purged from the alignment. Maximum-likelihood phylogenies of each alignment were constructed with RAxML [37], using the general time reversible (GTR) model of nucleotide substitution with a Gamma model of rate heterogeneity. Then, 100 bootstrap replicates were performed using the RAxML rapid bootstrapping algorithm [38]. The default values were used for all other options. The values of the Gamma distribution shape parameter alpha and the GTR nucleotide substitution rates were estimated from the data. Phylogenies were displayed using UGENE version 40.0, as phylograms with Bootstrap and distances displayed [39]. The closest available reference genome for each sequenced strain was identified using the 16S rRNA phylogeny and whole-genome comparisons were performed using NUCmer, part of the MUMmer package, using PATRIC version 3.6.11 (MinHash) [40]; and through MicrobesNG identifying the closest reference genome using Kraken [41, 42]. Genomic DNA analysis and sequence searching was performed using PATRIC and NCBI, while SignalP version 5.0 and SecretomeP version 2.0 were used to screen sequences for signal peptide presence [43–46].

### Lipase assays

Rhodamine B agar plates were used to indicate lipolytic bacterial colonies by the presence of lipid enzymes (lipase/esterase; [21]. To 1 l of autoclaved media agar (NB or SWWa) add 1 % (w/v) olive oil and 0.1 mg ml^−1^ Rhodamine B solution with vigorous shaking and the media poured into agar plates. Bacteria were streaked or spread onto these plates and lipolytic colonies were identified using a UV transilluminator and fluorescent colonies taken forward for further processing.

Lipase activity was also investigated semi-quantitatively using *p-*nitrophenol release from *p-*nitrophenol ester at 410 nm in a TECAN plate reader (INFINITE 2000). The reaction mixture contained 50 mM Tris-HCl pH 7.5, 1 mM CaCl_2_, 0.3 % (v/v) Triton X-100, 1 mM p-NPP, made fresh in every case, to 180 ul of this, 20 ul of bacterial supernatant was added. This was incubated at room temperature for 30 min and measuring at 410 nm. In our calculations we used the molar extinction coefficient reported for this substrate previously (17 300 M^−1^ cm^−1^ [47]; and report activity as nmoles pNP released per minute.

### Data availability

Sequence data generated in this study have been deposited to the European Nucleotide Archive (https://www.ebi.ac.uk/ena) with accession numbers as follows: *Serratia marcescens* SFB6, ERS4270774; *Klebsiella oxytoca* SFB9, ERS4270775; *Serratia liquefaciens* SFB10, ERS4270776; *Acinetobacter bouvetii* SFB21, ERS4270777; *Klebsiella pneumoniae* SFB23, ERS4270778; 16S sequencing data, ERS4556234. Raw data for FAME work is in Fig. A.1 and microbiome work is in Fig. A.3.

## Results

### Fatty acid profiling of fatberg samples

**Fig. 1.**
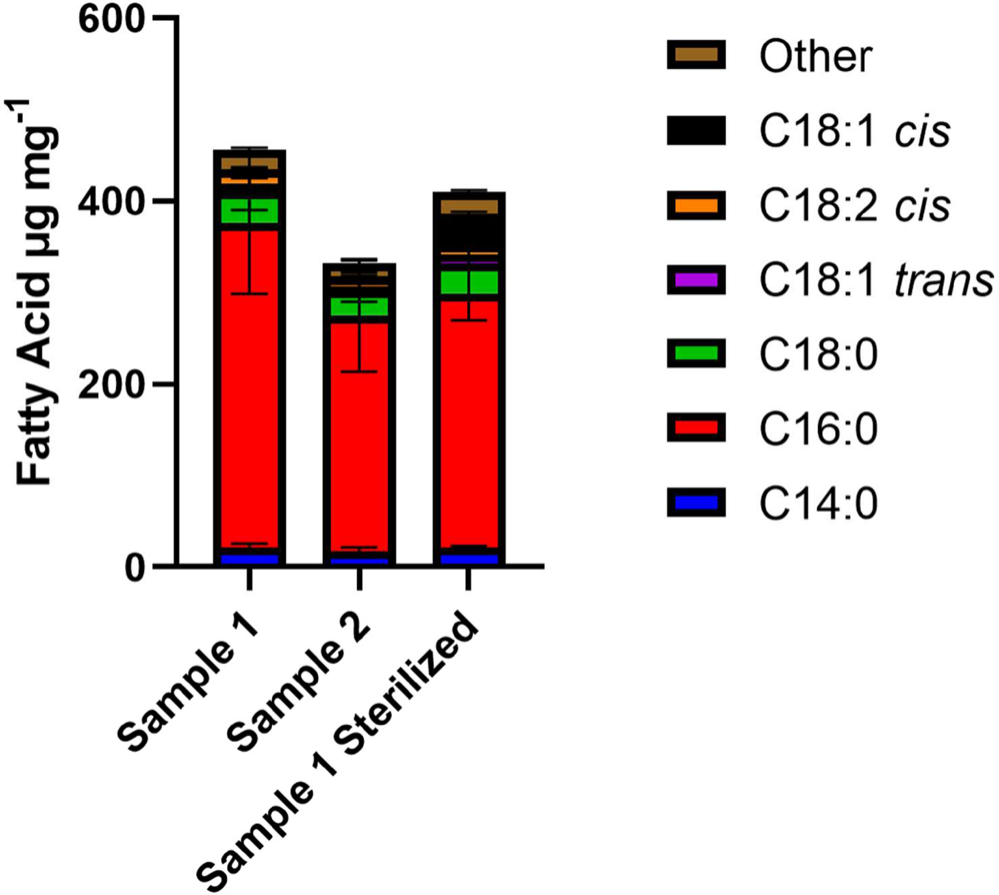
Bar charts showing the six most abundant fatty acids in two fatberg samples isolated from London sewers (‘sample 1’ and ‘sample 2’) and sterilized FOG sample isolated from sample 1 (‘sterilized sample 1’) as μg mg^−1^ Fatberg sample. Error bars show standard deviation.

In this paper, we began with one aim being to isolate a range of FOG degrading lipolytic strains from fatbergs within the wastewater environment using lipase activity as a screen. As a first step to ensure that our lipase screening methods would target the correct lipolytic profile in comparison to fatberg environments, we determined the lipid profile of solid fatberg samples taken from two separate exemplar Fatberg sites (site 1 and site 2) in London, UK, using gas chromatography-flame ionization detector (GC-FID) by comparison with known fatty acid methyl ester (FAME) standards on five samples from each fatberg. The data reveal that the fatbergs have different overall FOG content at 455.8 μg mg^−1^ and 331.9 μg mg^−1^ (Fig. 1, Table S1). However, in both cases the overall profiles (Fig. S1) were similar (not statistically different) with the most abundant fatty acid (FA) being C16 Palmitic acid (average 77 % of FAME present), followed by Myristic (C14, 5 %), stearic (C18, 8 %) and linoleic acids (C18 : 2 *cis*, 3.8 %). However, analysis of the minor constituent FAs revealed statistically significant differences in the amounts of *cis*-5,8,11,14,17-Eicosapentaenoic acid (C20 : 5; 0.776 % : 0.404 % *P*=0.0243), Behenic acid (C22; 0.478 %->0.282 % *P*=0.000752), (C24; 0.161 : 0.112 % *P*=0.00050.7) and Nervonic acid (C24 : 1; 0.200 % : 0.103 % *P*=8.61×10-5), however, given their low levels in the samples the importance of this is unclear. As part of our studies, we also sterilized a small portion of solid FOG sample from site 1 (in technical triplicate) via a dry-heat method (160** **°C, 2 h); the aim here was to assay any changes in the FAMEs profile and establish if the use of these samples in our enrichment and isolation experiments was possible. The heating process caused no significant overall change (*P*=0.364; paired *t*-test) when heat sterilized, however, a significant (*P*<0.05) increase in oleic acid (C18 : 1 *cis*; 0.2->8 % *P*=6.4×10^−5^) and also in amounts of Arachidonic acid (C20 : 4n6; 0.006 %->0.058 % *P*=0.0182) and *cis*-4,7,10,13,16,19-Docosahexaenoic acid (C22 : 6n3; 0.008 %->0.079 % *P*=0.031). At the same time, the amount of palmitate, the most abundant FA, reduced by 9.16 % but was not statistically significant (*P*=0.987).

### Isolation of lipase-producing bacteria from fatberg samples

To isolate potential FOG degraders from the blockage sites, wastewater, fatberg and sewer-wall swab samples were first pre-enriched in both a rich broth (Tryptic Soy Broth; TSB) and synthetic wastewater (SWWa) minimal media that contains a range of mineral salts, including acetate (14 mM, 0.81 mg l^−1^), a common wastewater carbon source [48, 49], with the addition of either olive oil (1 % v/v) or solid FOG sample (1 % w/v) and incubated for 3 days before being transferred to a new flask of either TSB or SWWa and enriched for 5 days at 15–20 °C [20]. Importantly this media has a C:N:P ratio of 100 : 5 : 1; which has been shown to be effective in environmental and lab studies to aid FOG degradation [50]. These enrichments were then spread onto both TSB and SWWa agar plates that also contained Rhodamine B (0.0001 %) and olive oil (1 %). Olive oil was chosen both for consistency but also due to its composition of oleic acid and linoleic (C18) and palmitic (C16) acid, long chain fatty acids all present in our fatberg (and heated) samples (Fig. 1) [21, 51].

**Fig. 2.**
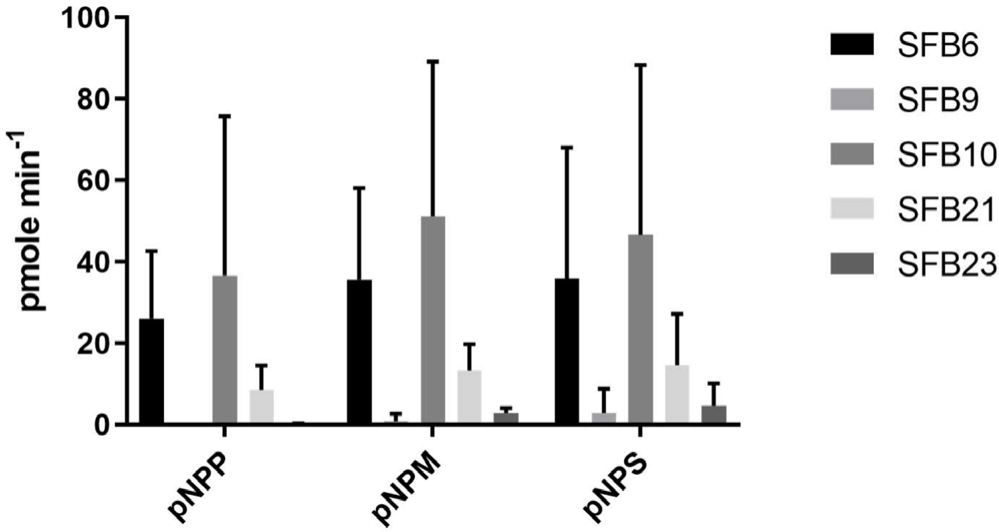
Bar chart to show the rate of activity in the presence of pNPP-Palmitate, -Myristate and -Stearate. Average of three cultures with sem shown.

In this method, lipolytic organisms cleave the FOG substrate, releasing FFA that then reacts with Rhodamine B, resulting in fluorescence that can be observed via ultraviolet illumination of the agar plates, with lipolysis appearing as orange fluorescent colonies (Fig. S2) [21, 51]. Using this method, colonies were identified with potential high lipolytic activity observed via the production of an orange halo on UV illumination and passaged on SWWa FOG and TSB plates. All five selected strains produced strong haloes on RhB agar (Fig. S2). In order to assess whether these strains contained secreted lipase activity they were grown in nutrient broth with 1 % olive oil, and culture supernatants (normalized to cell density) screened for activity against substrates representing the major lipid constituents in the FAME analysis, namely p-nitrophenyl-palmitate (pNP-P), pNP-myristate (pNP-M) and pNP-stearate (pNP-S). Using a combination of the RhB plates and pNP assays, we screened several hundred isolates and selected five with high lipase activity first by picking all colonies with Rh-haloes and then screening using the pNPP assay. These five isolates had lipase activity in the range up to 36.6 pmoles pNP released min^−1^, with activity against not only Palmitate but also Stearate and myristate (Fig. 2), validating our strategy of prescreening on RhB-oil plates. Of these, three were isolated with pre-enrichment in synthetic wastewater plus oil followed by TSB plus oil (21, 23), whilst two were pre-enriched in TSB plus oil followed by SWWa plus oil enrichment (6,9,10).

All five selected strains, from now on called SFB6, 9, 10, 21 and 23 (SFB: SheffieldFatBerg) displayed activity against pNP substrates. The results, reported in Fig. 2, showed that SFB6 and SFB10 had the highest activity against all substrates (25.8, 51.1 pmoles min^−1^ released) while SFB21 also had broad activity of 8.56–14.57 pmoles min^−1^ released for all substrates. Although SFB9 and 23 had clear haloes on RhB plates they exhibited significantly lower lipase activity under the conditions tested. None of the strains displayed a particular preference for any of the substrates over another, with the exception of SFB23, which seemed to prefer Stearate and Myristate over Palmitate.

### Genome analysis of isolates

Using a combination of 16S rDNA and whole-genome sequence phylogeny [MicrobesNG (Kraken) and PATRIC (MinHash)], the isolated SFB strains were identified as potential new strains of *Serratia marcescens* (SFB6)*, Klebsiella oxytoca* (SFB9)*, S. liquefaciens* (SFB10), *Acinetobacter bouvetii* (SFB21) and *K. pneumoniae* (SFB23) (Table S3*,* Fig. S3). Notably, these bacterial isolates are all common environmental organisms that have been reportedly found in soil, water sources and treatment plants. In order to characterize these organisms further, we examined their genome sequences for the presence of potential lipase enzyme encoding and lipid transport and metabolism genes. Using Illumina-based sequencing each genome dataset was assembled into contigs and submitted to the European Nucleotide Archive (ENA; see Table 1 for accession numbers).

**Table 1.** Summary genome information including accession (Ac) number

Isolate	Homology	#Contigs	No. of Bases	Size (Mb)	G+C content (%)	Ac no.
SFB6	Serratial marcescens	77	332.8	5.339	59.18	ERS4270774
SFB9	Klebsiella oxytoca	155	665.6	6.38	55.31	ERS4270775
SFB10	Serratia liquefaciens	29	290	5.2	55.36	ERS4270776
SFB21	Acinetobacter bouvetti	69	253.8	3.46	51.24	ERS4270777
SFB23	Klebsiella pneumoniae	47	156.4	5.42	57.22	ERS4270778

**Fig. 3.**
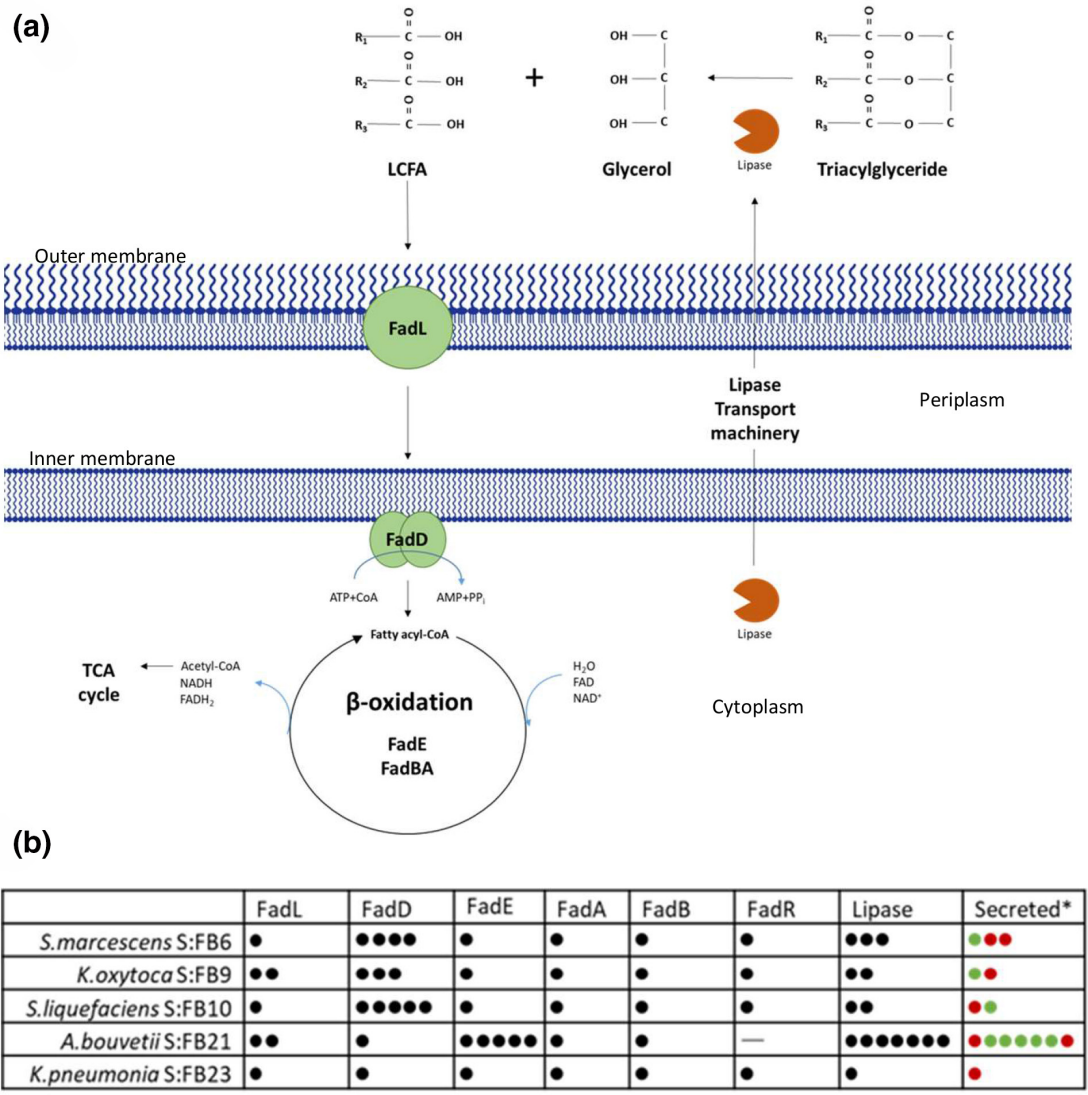
(a) Illustration to show the bacterial FOG catabolism pathway (adapted from [92]). FadL, long-chain fatty acid transport protein; FadD, fatty acid CoA ligase; FadE, acyl-CoA dehydrogenase; FadA, 2-ketoacyl-CoA thiolase; FadB, enoyl-coA hydratase; LCFA, long-chain fatty acid. (b) Summary of homologous genes to bacterial lipid catabolism genes and lipases in SFB isolate strains. Dots correspond to homologous genes in sequence, green represents presence of transport sequence detected. Genomic DNA analysis and sequence searching was performed using PATRIC and NCBI, predicted secreted genes generated using SignalP and SecretomeP (see Methods).

All of the five bacterial isolates contain at least one putative lipase encoding gene sequence, (Fig. 3b). Despite the lipolytic activity, SFB23 (* K. pneumoniae *) does not appear to contain a putative secreted lipase sequence in its genome sequence, implying that it is not exported via a standard secretion signal or that other unknown exported lipases exist in this strain. Of the strains isolated *Acinetobacter* SFB21 has the highest number of putative lipase sequences, with seven putative lipase genes of which five are predicted to be secreted enzymes (PSORT), with all lipases containing predicted active sites matching those of the GXXX family [52].

The fatty acid degradation pathways and the main proteins involved in the lipid metabolism have been highlighted in Fig. 3a), i.e. to establish the likelihood these organisms can utilize as well as produce FFAs. All isolates contain full putative fatty acid degradation pathways (Fig. 3b) and the predicted genes encoding fatty acid transport (*fadL* and *fadD*), the β-oxidation pathway for fatty acid metabolism (*fadA*, *fadB* and *fadE*) alongside the transcriptional repressor (*fadR,* whose repression is relieved by fatty acid binding to the protein). All five isolates contained at least one copy of each of the genes apart from *A. bouvetii* SFB21, which did not contain a recognisable *fadR* gene – indicating that canonical fatty acid regulated control of gene expression of the β-oxidation genes is absent – i.e. it might be potentially constitutively expressed. On the other hand, SFB21 also contained multiple copies of FadE, which may indicate the ability to process FAs of a broad range. Both of the *Serratia* species contained multiple *fadD* genes – encoding the Acyl-CoA synthetase (FadD), which activates the fatty acid for entrance into the β-oxidation cycle, but which most likely encode enzymes with specificities for small, medium or long-chain fatty acids, which may enable more efficient transport of a range of fatty acids into the cell.

### Growth of SFB isolates on FOGs

**Fig. 4.**
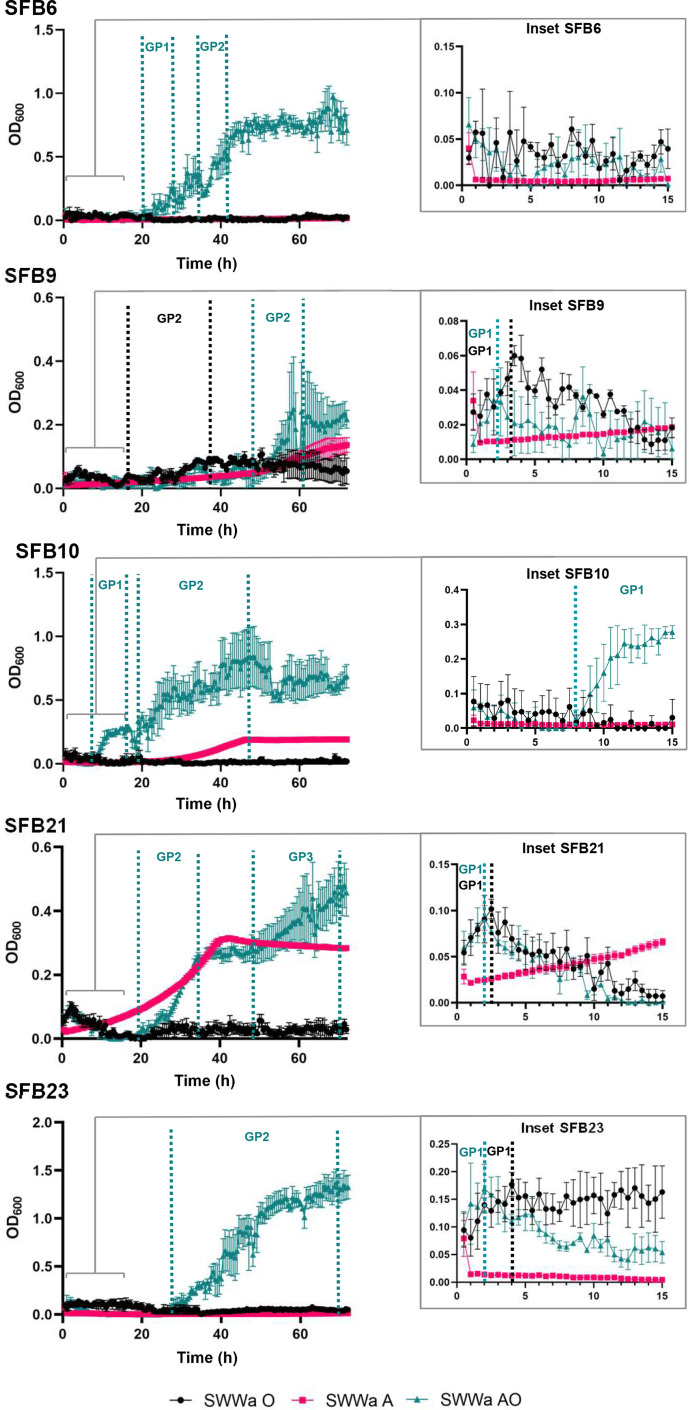
Growth curves of the five isolates in SWWa with olive oil (SWWa +oil, black), acetic acid (SWWa A, magenta) or both olive oil and acetic acid (SWWa A+oil, teal). Vertical lines in the relevant colours and accompanied by GP1/2/3 depict putative alternate growth phases. The inset depicts the growth curve in the first 15 h in more detail. These are representative graphs of three technical replicates that were repeated three times. The OD_600_ is plotted with error bars showing standard error of the mean.

The five strains were then tested for their ability to grow in synthetic wastewater with either addition of FOG (1 % olive oil) or FOG plus acetate (14 mM, 0.81 mg ml^−1^). The strains were grown at 25C in a Tecan Sunrise with horizontal shaking with OD_600_ measurements taken at 30 min intervals, and all wells having respective control wells containing appropriate media blanks for each condition, which were subtracted from the culture positive wells to rule out any emulsification or precipitation effects. As shown in Fig. 4 we incubated the cultures for 72 h with all strains displaying multiphasic growth patterns. In general, all isolates grew in the presence of acetate plus olive oil (Fig. 4, green) with varying peak OD_600_ (0.24 to 1.4).

Isolates SFB 9, and 21 show growth in the presence of acetic acid only (magenta) with increased growth or altered growth rate in the presence of olive oil and acetic acid (teal, SFB9 : 0.24 vs 0.15; SFB21 : 0.48 vs 0.31), whereas no growth on acetate alone was observed for isolates SFB 6, 10 and 23 (Fig. 4). Growth on olive oil only was generally low for all isolates compared to olive oil plus acetate but some growth was observed.

To illustrate this, we highlight the first 15 h in an inset in Fig. 4, revealing that for SFB 9, 10, 21 and 23 growth characteristics are different in the first 8 h as compared to extended 72 h incubation (Fig. 4 insets). For example, analysing growth on oil, cultures displayed a short rapid period of growth within the first 5 h with OD_600_ reaching around 0.06–0.18 for SFB 9, 10, 21 and 23 (Fig. 4, insets GP1, black), which tails off, except for SFB9, which maintains slow growth on oil for 50 h (GP2, black) . In the case of the acetate +oil cultures, all strains display a bi- or even tri-phasic growth pattern, where initial and subsequent growth periods are followed by periods of potential quiescence or adaptation before OD_600_ rises again, illustrated in Fig. 4 (GP2/3, teal), with isolate SFB21 a prime example. These data indicate a potential switch in growth modes/substrate or accumulation of toxic compounds in the cultures or possibly indicating alternating carbon sources that might arise from sequential FOG degradation – though this would need further investigation.

Taken together these data indicate the ability of these strains to grow in synthetic wastewater both in the presence of the FOG substrate olive oil but that they require acetate to boost growth to higher levels.

### 16S rDNA microbiome analysis of Fatberg site

In order to characterize the microbial community present and ascertain if representatives of our isolates were present at the FOG blockage sites or might even be dominant, 16S rDNA sequencing was carried out to determine the resident environmental bacterial microflora. In total, three swabs were taken from FOG deposits at site 1 and site 2 and the DNA extracted from the swabs using DNeasy PowerSoil kit and the V3/V4 variable regions were sequenced [commercially at MrDNA using primer set 341 F/ 806R [25]; on an Illumina MiSeq (MR DNA, Shallowater, TX, USA]. In these samples an average of 285 OTUs (+/-67 sd) were detected (Table S3).

**Fig. 5.**
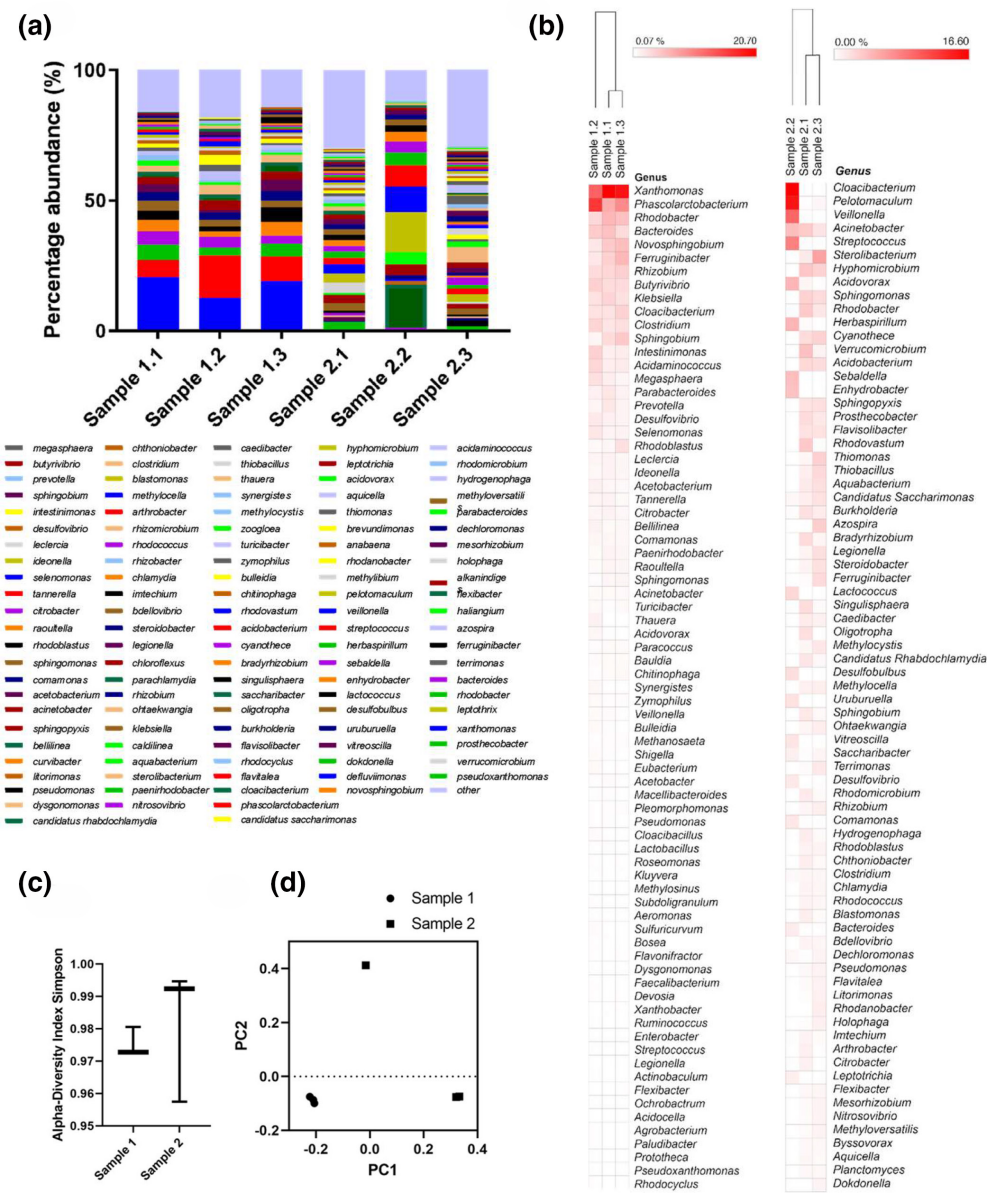
(a) Bar charts showing the genus representing >/=0.5 % found by 16S NGS sequencing from two FOG blockage sites in London sewers. (b) Heatmap displaying the top 75 most frequent genera based on the number of reads per sample (generated using Morpheus). (c) Simpson’s index alpha diversity analysis of the sequencing data showing *P*-value: 0.66099; [T-test] statistic: −0.50313. (d) Principal coordinate analysis (PCoA) based on Jensen–Shannon divergence distance showing similarities of samples from two fatberg sampling sites. PERMANOVA [f-value: 5.2369; R-squared: 0.56695; *P*-value<0.1].

The genus level data representing genera present at 0.5 % of total reads or above for each sample are shown in Fig. 5a, and the top 75 most frequent genera detected of the total genus reads displayed by heatmap (Fig. 5b). Alpha diversity analysis of the samples (Simpson’s index) is shown in Fig. 5c and varies between the sample groups, but the significance of the analysis is limited by the small sample size. A principal coordinates' analysis (Jansen–Shannon PCoA; Fig. 5d) does not show significant clustering of samples between sites 1 and 2, but does highlight that sample site 1 samples cluster together closely, however, sample site 2 has a larger diversity between its samples, with sample 2.2 clearly being more of an outlier in this analysis – again we add the caveat of small sample sizes here.

Our data reveal that these fatberg microbiota are composed of a diverse range of aerobic and anaerobic bacteria, with many of these genera containing species that are known to degrade lipids and commonly found in wastewater systems, e.g. *Xanthomonas* and *Rhodobacter* [53–55]. At site 1, *Xanthomonas* is at the highest levels in all three samples (12–20 %) with the anaerobe *Phascolarcobacterium* (6–16 %) the next most abundant. Notably several other anaerobes are present, such as *Bacteroides* (2.9–5 %), *Clostridium* (2.2–3.4 %), *Cloacibacterium* (2.25–3.6 %) as well as *Selenomonas *, *Prevotella *, *Parabacteriodes* and *Tannerella *, which are all part of the anaerobic gut flora, but more broadly indicate the presence of anaerobic micro-environments in these FOG deposits. Notably the facultative genus *Klebsiella* (2.4–3.6 %) were also present at significant levels. Sample 2 is much more variable with *Cloacibacterium* at a very high level in sample 2.2 (16.6 %) but below 0.2 % in sample 2.1 and 2.3. The sequence with highest median value across the sample 2 datasets here is *Acinetobacter* (1.7–4.2 %), of note given our isolation data; and followed by the common environmental organism *Hyphomicrobium* (0.03–3.6 %). However, sample 2 has no dominant organism and a large quantity of sp. at low levels, indicating high diversity. Most of the genera mentioned above are common across both locations, with it notable that *Acinetobacter, Klebsiella* and *Serratia* as well as several environmental (* Rhizobium *), gut (*Prevotella, Tannerella, Citrobacter* and *Bacteroides *) were among these.

## Discussion

In this study, we set out to establish a pipeline for the isolation of potential fatberg-specific biodegraders and better understand the microbial environment of fatbergs to pave the way towards more target environment tailored products in the future.

As the first part of the study, we observed that the dominant fatty acid found in two separate fatberg deposits was palmitic acid (75 % of the whole sample) – a finding that complements findings of other researchers in the USA and UK [4, 8, 56].

We then developed a strategy that utilized high-throughput screening on Rhodamine agar plates, before secondary screening using a palmitate based colorimetric substrate (the major fatty acid in our samples) in a microplate format. In our growth experiments, isolates SFB9, 10 and 21 were able to grow on acetate only with isolates SFB6 and 23 showing no observable growth. All of the isolate growth seemed to be boosted by FOG (olive oil) inclusion in the media but only when acetate was present, with lower growth observable with olive oil as the only carbon source, thus highlighting their ability to potentially degrade and utilize FOG as a substrate for growth. This is reminiscent of diauxic growth that is displayed by a range of organisms when switching carbon sources [57]. While other organisms often grow on multiple substrates, as was shown with *Pseudomonas aeruginosa* in the context of growth in tap water [58]. Finally, starvation in cultures can often influence metabolic capacity with glucose starved lipolytic *B. licheniformis* showing upregulation of lipid degrading pathways [59]. Considering our enrichment media contained acetate as well as FOG, a potential diauxie (e.g. SFB21) is perhaps not unexpected and our future studies will examine how varying acetate levels affects FOG-dependent growth and lipase production of isolates and indeed FOG levels in the media – assayed using Gravimetric methods. Notably, in preliminary studies (unpublished data), we observed a reduction in FOG levels in spent media when all five bacteria were grown in a consortium for 48 h with most degradation occurring later in the incubation cycle. One other aspect for development would be the enrichment strategy itself, with a reduction in acetate levels potentially allowing selection of organisms more dependent on FOG and allow assessment of the impact of alternative carbon sources in media on FOG metabolism or taking a limiting dilution approach as has been used in other environments to potentially improve recovery of organisms [60]. For example, there is evidence that some organisms upregulate lipase production in response to other carbon sources, with *Acinetobacter iwoffii,* which is related to SFB21, increasing lipase production in the presence of other hydrocarbons [61].

After genome sequencing, it was evident that our isolated strains contained a complement of predicted fatty acid degradation (*fad*) genes, containing uptake and catabolism capability as well as a complement of lipases (Fig. 3b). These include lipase enzymes that are putatively secreted from the cell to hydrolyse TAGs (Triacylglycerols) into long-chain fatty acids (LCFAs) and a glycerol backbone. The LCFAs are transported into the cell using FadL transporter [62] and converted into coenzyme A (CoA) thioesters by the inner-membrane associated FadD, activating them [63]. This then enters the β-oxidation cycle where FadE converts acyl-CoA to enoyl-CoA and then FadBA tetramer catalyses the hydration, oxidation generating NADH and FADH2 and finally shortening of the acyl-CoA to give acetyl-CoA, which is processed by the TCA cycle [64]. The genomics of our strains identified two *Serratia *, two *Klebsiella* and a non-pathogenic *Acinetobacter* strain.

Of these strains, there have been several reports of stable lipase enzymes, with those from the *Serratia* strains the most well characterized, e.g. LipA from *S. marcesens* and SlLipA form *S. liquefaciens* [65, 66]. Similarly, interest in lipase from *Klebsiella pneumoniae* and *oxytoca* strains in relation to biodiesel production has also been reported with strains that were isolated from restaurant wastewater identified as degrading FOG [65–69]. In the case of *Acinetobacter* SFB21, this is part of a genus in which there is biotechnological interest with several novel lipases now discovered [70]. Notably SFB21 is most closely related to non-pathogenic *Acinetobacter* spp. that includes *A. schindleri *, *bouvetii, Iwoffi* and *johnsonii* and thus may have the potential for use in scale-up for deployment [71–73] – N.B. a future focus of our work to determine whether SFB21 is a novel *Acinetobacter* spp. In fact, *Acinetobacter* strains, including SFB21 contain multiple copies of the *fadE* gene in their genomes, indicating a broad lipid catabolic capability [74], while *Acinetobacter* strains are known to grow well on acetate and palmitate [75–78], and to produce internal lipid-bodies for energy storage [79, 80]. One future focus however will be to understand under what conditions the Fad genes are expressed, i.e. in the design of environmental deployment strategies within products- such as feedstocks.

Finally, *Acinetobacter* strains have been considered for biotechnological applications (including biofuel, pharmaceuticals and cosmetics) for the conversion of carbon substrates into useful oils such Triacylglycerols or in the form of Polyhydroxy Alkanoate polymers [81, 82]. This indicates the potential ability to use *Acinetobacter* strains (including SFB21) to remove FOG from a system, a capability that would be useful in FOG remediation applications and may suggest that future isolation strategies may consider targeting *Acinetobacter* strains more specifically using specialised media [83].

While the isolated strains have potential for remediation, one clear concern is potential pathogenic capability. This is a difficult conundrum since unsurprisingly these pathogenic faecal organisms are present in wastewater, however, it is in manufacturing processes and facilities that a potential problem arises with the manufacturing of potentially pathogenic *Klebsiella* and *Serratia* strains at scale. Therefore, given the non-pathogenic nature of *Acinetobacter schindleri* and *bouvetii* strains [77], like SFB21 here, it may be that future strategies should concentrate on the isolation of FOG degrading *Acinetobacter* strains for use in microbial consortia for FOG degradation as an alternative to *Bacillus* and *Pseudomonas* strains, although we acknowledge much of this work focuses on grease traps [84].

Finally, fatberg deposits are not composed solely of FOG, but are also made up of proteinaceous and carbohydrate substances [85]. Hence it is likely that any useful bioaddition strains or consortia should also have the ability to degrade a range of substrates. It is thus notable that the isolates in our study are also known for their ability to grow on a range of substrates and produce extracellular proteases, as seen in our genome sequences (not shown) but also in the literature [86].

As with any culture-based enrichment or selection strategy, the snapshot of organisms present is biassed by that enrichment media. Therefore, we also conducted 16S rDNA metagenomics of our two fatberg samples using Illumina-based sequencing. Firstly, the genus *Acinetobacter* and *Klebsiella* were found across all samples in the 16S rDNA sequencing study performed (ranging from 0.36–4.2% and 0.04–3.6%, respectively). In contrast, *Serratia* was only detected as a minor component of the fatberg microbiome (<0.01 %). Of note, the genera commonly used in FOG biological treatment products are reported to be *Pseudomonas* and *Bacillus* species [84, 87], but these contributed to less than 1 % of the genera in our NGS screens. These data also revealed a diverse microbial community in these samples, on average around 300 potential species, many of which are commonly seen in wastewater samples (e.g. *Xanthomonas *, Fong and Tan [88]; *Sphingomonas *, Yoon *et al*. [89]; Rhodoacter, Hiraishi *et al.* [90]). In one of the sample site datasets (sample 1); there was dominance by the genus *Xanthomonas* (17.5 % of reads), an organism well known for its lipolytic properties in the context of plant pathogenesis [91]. In contrast, data from sample 2 were more variable, containing many spp. at much lower levels. Perhaps surprisingly both contained a significant number of obligate anaerobes, many members of the gut microflora (e.g. *Bacteroides *, *Cloacibacterium *, *Prevotella *) – however, our enrichment process would of course select against these and again may suggest future work should possibly take this into account. Future studies incorporating anaerobic selection conditions, alternative carbon sources (e.g. other carbohydrates, e.g. starch [85]) or limiting dilution approaches may improve recovery of species [60].

As outlined we chose five highly active strains for further characterization by genome sequencing and in growth experiments. Notably, all of our strains showed growth in the presence of olive oil as a FOG substrate in a synthetic wastewater medium (Fig. 4), which was boosted by inclusion of acetate indicating the potential to both degrade and utilize FOGs in the environment. While we have successfully isolated a range of FOG utilizing lipolytic strains, we are aware that none of our strains are well-adapted to growth solely on FOG, indicating that any potential application will need to ensure the presence of acetate. Alternatively, future enrichment strategies should attempt to isolate strains without acetate or attempt to adapt or evolve strains to improve FOG-dependent growth/degradation. Favourably, acetate levels are known to be significant in wastewater [48], suggesting that these organisms should survive *in situ* and that co-inoculation with acetate-based media may boost lipolysis. Carbon and nitrogen ratios have been identified as important for lipid degradation and so further investigation to determine performance and application of FOG degrading consortia would be important for future development [85].

### Conclusion

In this study, we have increased understanding of the environment at FOG blockages and improved our understanding of microbial communities (microbiome) at fatberg sites, identifying for the first time the diverse range of organisms present.

Additionally, our isolation of a selection of lipolytic strains able to utilize FOGs as growth substrates highlights the potential of this approach of going to the environment for natural tailored solutions to solid FOG blockages, especially since they differ from current bioaddition strains in use (e.g. *Bacillus* strains). We consider these organisms, and especially *Acinetobacter* strain SFB 21 as having potential as part of a FOG degrading consortia, and preliminary unpublished work indicates that they are capable of reducing FOG content in domestic wastewater under laboratory conditions, and will be a focus of future work. We, therefore, have devised a strategy that could be applied to other wastewater situations to isolate potential FOG-treatment strains that are currently being taken forward and pave the way for the production of FOG-blockage tailored microbial consortia targeting solid deposits that may outperform current solutions.

## Supplementary Data

Supplementary material 1

Supplementary material 2
