# Supplementary material for: Looking through the FOG: microbiome characterization and lipolytic bacteria isolation from a fatberg site
Source: Microbiology (Reading). 2021 Dec 6;167(12):001117. doi: 10.1099/mic.0.001117 (PMC8744997; doi:10.1099/mic.0.001117)
Supplement: Supplementary material 1 [file mic-167-1117-s001.pdf]

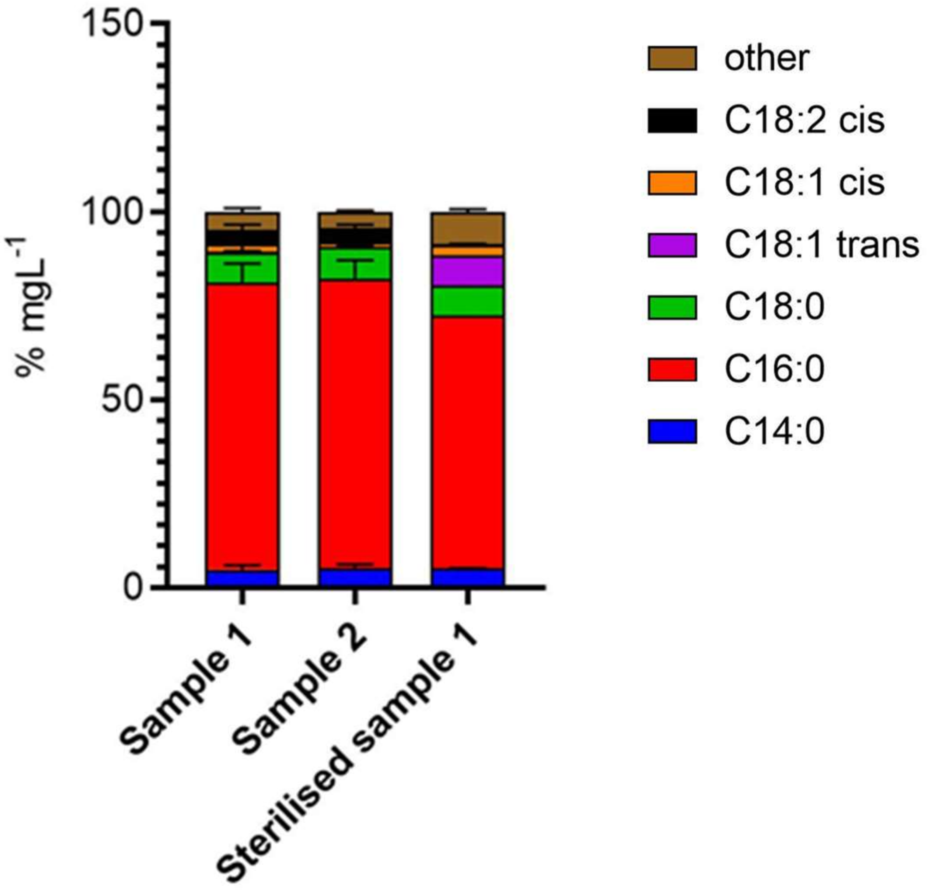

Figure S1 Bar charts showing the 6 most abundant fatty acids in 2 fatberg samples isolated from London sewers (“Sample 1” and “Sample 2”) and sterilised fog sample isolated from Sample 1 (“Sterilised sample 1”) as percentage abundance of fatty acids in Fatberg sample. Error bars show standard deviation.

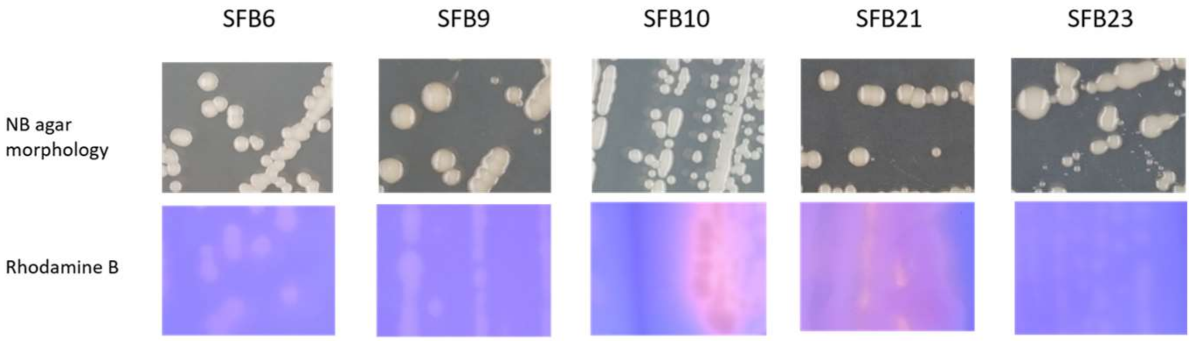

Figure S2: Images of SFB isolates on TSB agar (top row) and under UV illumination on SWWa plates containing olive oil (1%) and Rhodamine B (second row).

Figure S3: Phylogenetic trees for the five strains isolated in this study – trees generated as outlined in the methods section (MUSCLE, RaXML) and displayed as Phylograms with bootstrap values on nodes, distances shown.

A

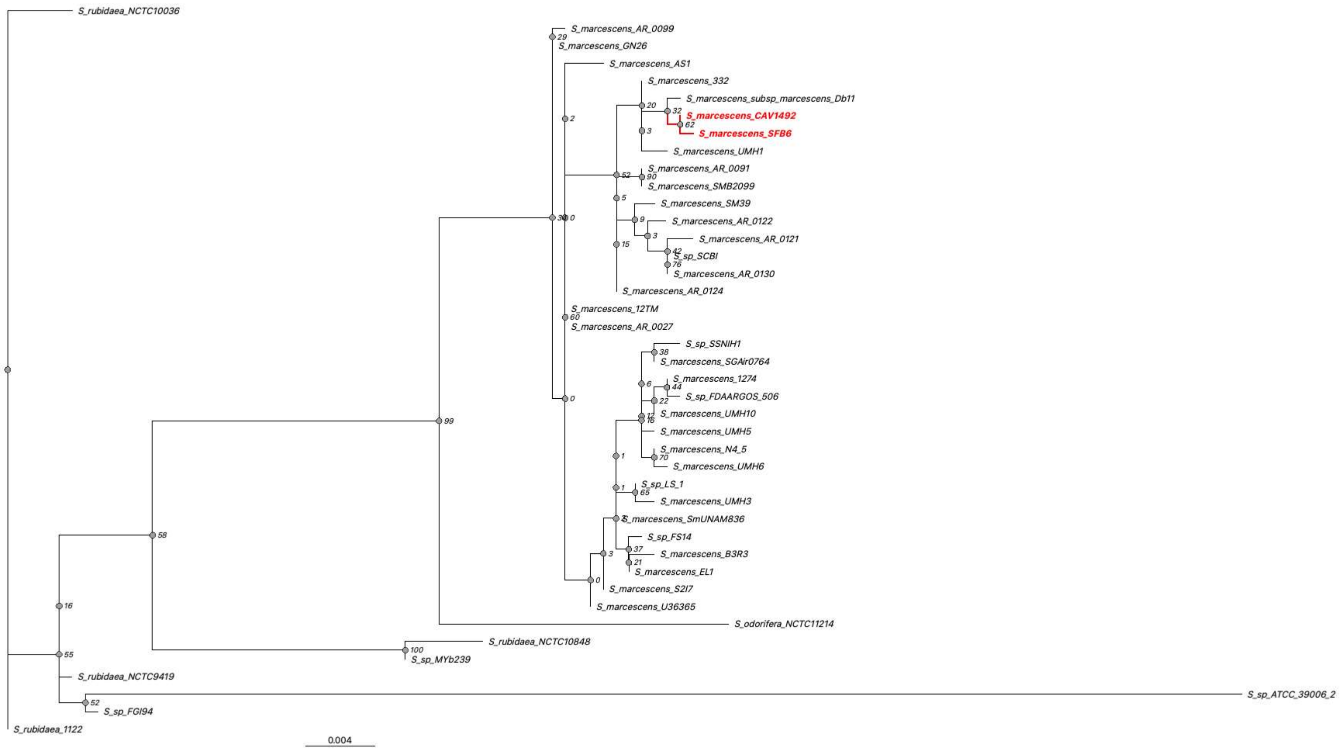

B

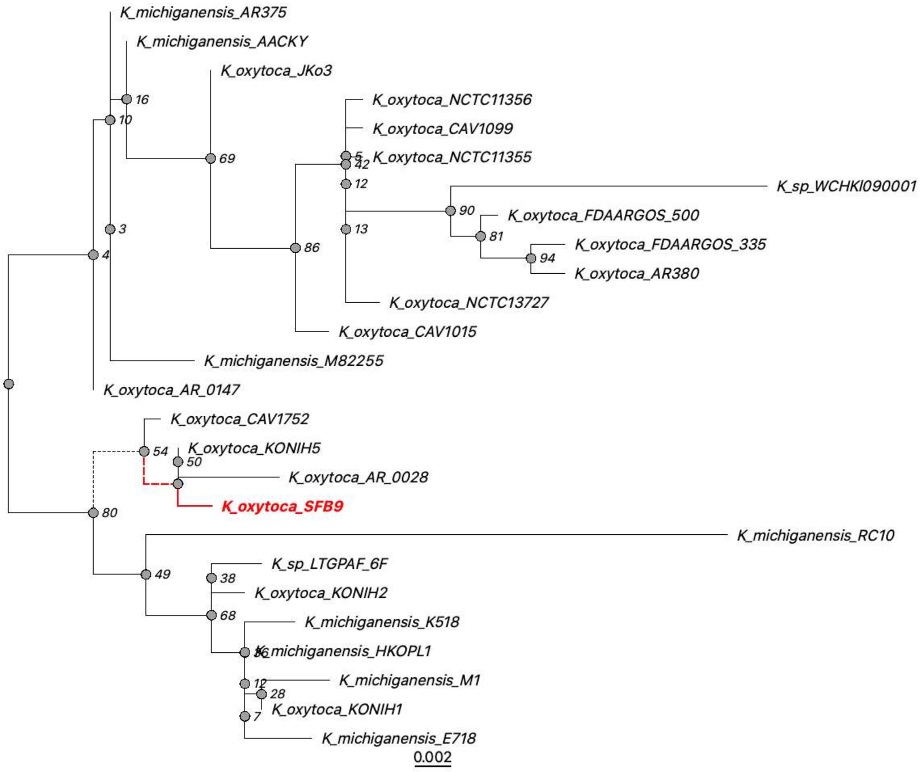

C

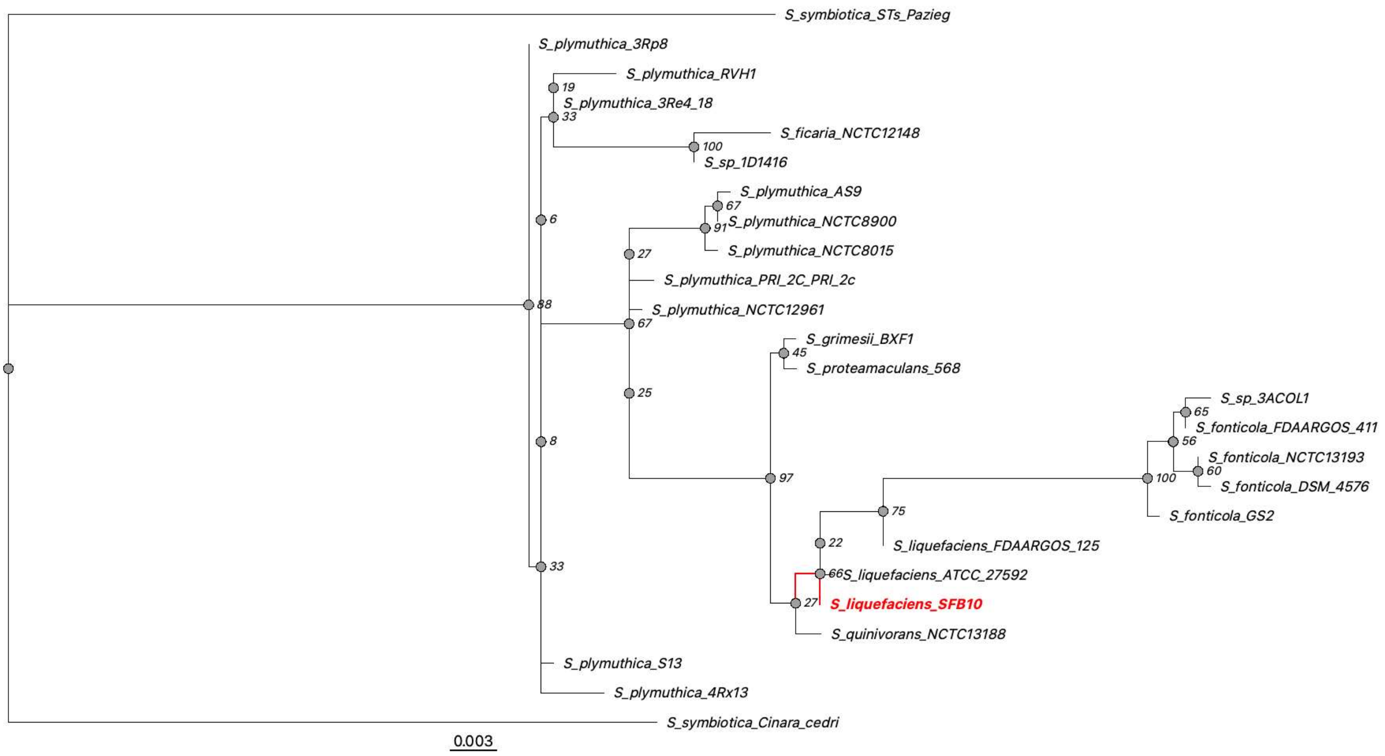
S_symbiotica_STs_Pazieg
S_plymuthica_3Rp8
S_plymuthica_RVH1
S_plymuthica_3Re4_18
S_ficaria_NCTC12148
S_sp_1D1416
S_plymuthica_AS9
S_plymuthica_NCTC8900
S_plymuthica_NCTC8015
S_plymuthica_PRI_2C_PRI_2c
S_plymuthica_NCTC12961
S_grimesii_BXF1
S_proteamaculans_568
S_sp_3ACOL1
S_fonticola_FDAARGOS_411
S_fonticola_NCTC13193
S_fonticola_DSM_4576
S_fonticola_GS2
S_liquefaciens_FDAARGOS_125
S_liquefaciens_ATCC_27592
S_liquefaciens_SFB10
S_quinivorans_NCTC13188
S_plymuthica_S13
S_plymuthica_4Rx13
S_symbiotica_Cinara_cedri
0.003

D

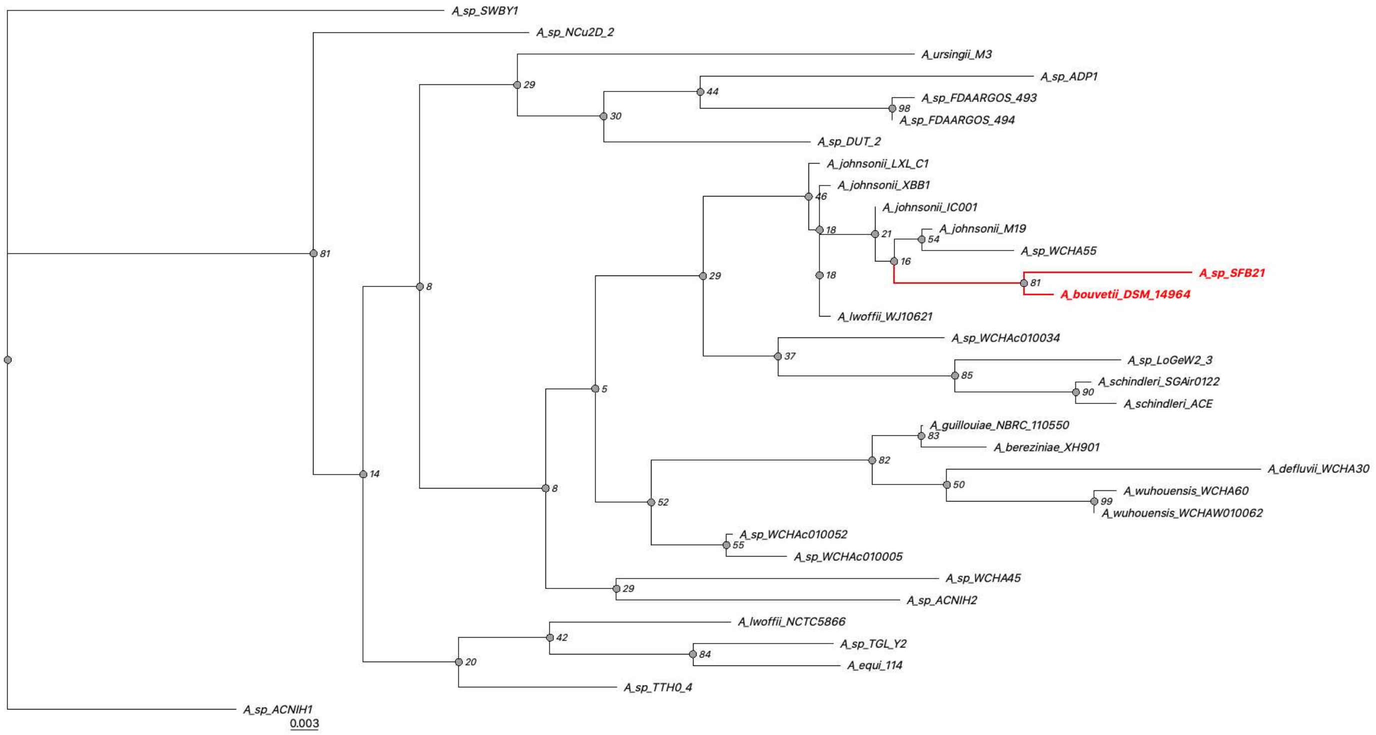
A_sp_SWBY1
A_sp_NCu2D_2
A_ursingii_M3
A_sp_ADP1
A_sp_FDAARGOS_493
A_sp_FDAARGOS_494
A_sp_DUT_2
A_johnsonii_LXL_C1
A_johnsonii_XBB1
A_johnsonii_IC001
A_johnsonii_M19
A_sp_WCHA55
A_sp_SFB21
A_bouvetii_DSM_14964
A_lwoffii_WJ10621
A_sp_WCHAc010034
A_sp_LoGeW2_3
A_schindleri_SGAir0122
A_schindleri_ACE
A_guillouiae_NBRC_110550
A_bereziniae_XH901
A_defluvii_WCHA30
A_wuhouensis_WCHA60
A_wuhouensis_WCHAW010062
A_sp_WCHAc010052
A_sp_WCHAc010005
A_sp_WCHA45
A_sp_ACNIH2
A_lwoffii_NCTC5866
A_sp_TGL_Y2
A_equi_114
A_sp_TTH0_4
A_sp_ACNIH1
0.003

E

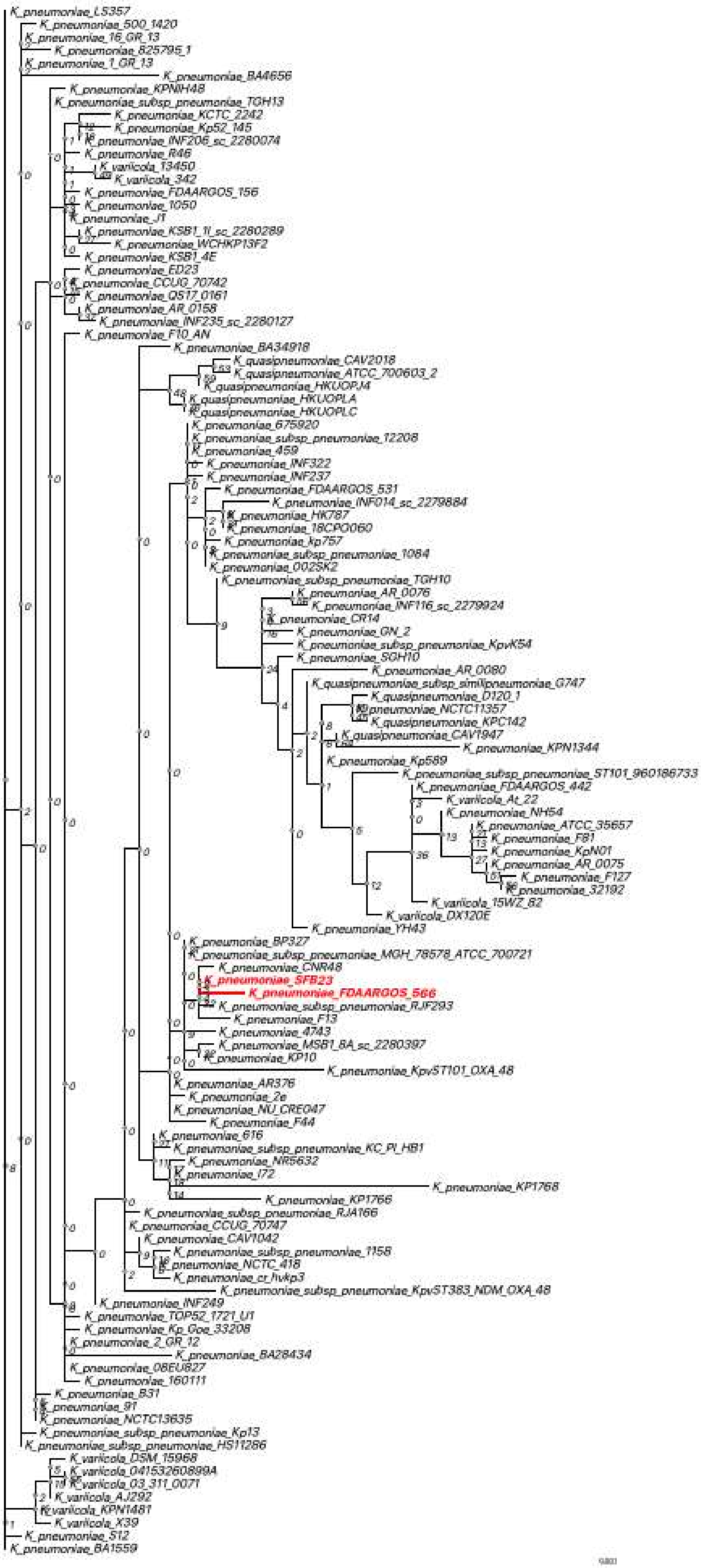

K_pneumoniae_LS357
K_pneumoniae_500_1420
K_pneumoniae_16_GR_13
K_pneumoniae_825795_1
K_pneumoniae_1_GR_13
K_pneumoniae_BA4656
K_pneumoniae_KPNIH48
K_pneumoniae_subsp_pneumoniae_TGH13
K_pneumoniae_KCTC_2242
K_pneumoniae_Kp52_145
K_pneumoniae_INF206_sc_2280074
K_pneumoniae_R46
K_varicola_13450
K_varicola_342
K_pneumoniae_FDAARGOS_156
K_pneumoniae_1050
K_pneumoniae_J1
K_pneumoniae_KSB1_1I_sc_2280289
K_pneumoniae_WCHKP13F2
K_pneumoniae_KSB1_4E
K_pneumoniae_ED23
K_pneumoniae_CCUG_70742
K_pneumoniae_QS17_0161
K_pneumoniae_AR_0158
K_pneumoniae_INF235_sc_2280127
K_pneumoniae_F10_AN
K_pneumoniae_BA34918
K_quasipneumoniae_CAV2018
K_quasipneumoniae_ATCC_700603_2
K_quasipneumoniae_HKUOPJ4
K_quasipneumoniae_HKUOPLA
K_quasipneumoniae_HKUOPLC
K_pneumoniae_675920
K_pneumoniae_subsp_pneumoniae_12208
K_pneumoniae_459
K_pneumoniae_INF322
K_pneumoniae_INF237
K_pneumoniae_FDAARGOS_531
K_pneumoniae_INF014_sc_2279884
K_pneumoniae_HK787
K_pneumoniae_18CPO060
K_pneumoniae_kp757
K_pneumoniae_subsp_pneumoniae_1084
K_pneumoniae_002SK2
K_pneumoniae_subsp_pneumoniae_TGH10
K_pneumoniae_AR_0076
K_pneumoniae_INF116_sc_2279924
K_pneumoniae_CR14
K_pneumoniae_GN_2
K_pneumoniae_subsp_pneumoniae_KpvK54
K_pneumoniae_SGH10
K_pneumoniae_AR_0080
K_quasipneumoniae_subsp_similipneumoniae_G747
K_quasipneumoniae_D120_1
K_pneumoniae_NCTC11357
K_quasipneumoniae_KPC142
K_quasipneumoniae_CAV1947
K_pneumoniae_KPN1344
K_pneumoniae_Kp589
K_pneumoniae_subsp_pneumoniae_ST101_960186733
K_pneumoniae_FDAARGOS_442
K_varicola_At_22
K_pneumoniae_NH54
K_pneumoniae_ATCC_35657
K_pneumoniae_F81
K_pneumoniae_KpN01
K_pneumoniae_AR_0075
K_pneumoniae_F127
K_pneumoniae_32192
K_varicola_15WZ_82
K_varicola_DX120E
K_pneumoniae_YH43
K_pneumoniae_BP327
K_pneumoniae_subsp_pneumoniae_MGH_78578_ATCC_700721
K_pneumoniae_CNR48
K_pneumoniae_SFB23
K_pneumoniae_FDAARGOS_566
K_pneumoniae_subsp_pneumoniae_RJF293
K_pneumoniae_F13
K_pneumoniae_4743
K_pneumoniae_MSB1_8A_sc_2280397
K_pneumoniae_KP10
K_pneumoniae_KpvST101_OXA_48
K_pneumoniae_AR376
K_pneumoniae_2e
K_pneumoniae_NU_CRE047
K_pneumoniae_F44
K_pneumoniae_616
K_pneumoniae_subsp_pneumoniae_KC_Pl_HB1
K_pneumoniae_NR5632
K_pneumoniae_I72
K_pneumoniae_KP1768
K_pneumoniae_KP1766
K_pneumoniae_subsp_pneumoniae_RJA166
K_pneumoniae_CCUG_70747
K_pneumoniae_CAV1042
K_pneumoniae_subsp_pneumoniae_1158
K_pneumoniae_NCTC_418
K_pneumoniae_cr_hvkp3
K_pneumoniae_subsp_pneumoniae_KpvST383_NDM_OXA_48
K_pneumoniae_INF249
K_pneumoniae_TOP52_1721_U1
K_pneumoniae_Kp_Goe_33208
K_pneumoniae_2_GR_12
K_pneumoniae_BA28434
K_pneumoniae_08EU827
K_pneumoniae_160111
K_pneumoniae_B31
K_pneumoniae_91
K_pneumoniae_NCTC13635
K_pneumoniae_subsp_pneumoniae_Kp13
K_pneumoniae_subsp_pneumoniae_HS11286
K_varicola_DSM_15968
K_varicola_04153260899A
K_varicola_03_311_0071
K_varicola_AJ292
K_varicola_KPN1481
K_varicola_X39
K_pneumoniae_512
K_pneumoniae_BA1559

Table S2: Summary genome information for isolated strains.

	Data Summary				
Isolate	Median insert size	Mean coverage	Mean coverage excluding 0s	Number of reads	Number of reads w/ insert size > 300
SFB6	616	95.0336	95.0343	1147901	774103
SFB9	540	162.92	162.93	2352305	1534163
SFB10	373	87.5434	87.5434	1159183	600196
SFB21	375	113.124	113.13	902043	99221
SFB23	643	43.774	43.7752	542515	375912

	Assembly and Annotation											
Isolate	# contigs (>= 0 bp)	# contigs (>= 1000 bp)	Total length (>= 0 bp)	Total length (>= 1000 bp)	# contigs	Largest contig	Total length	GC (%)	N50	N75	L50	L75
SFB6	183	70	5387953	5339387	77	404067	5343741	59.18	143660	108178	12	22
SFB9	299	134	6444530	6375077	155	681646	6390198	55.31	195792	97345	11	24
SFB10	112	21	5243152	5205013	29	1384747	5209645	55.36	649002	293920	3	6
SFB21	132	64	3491448	3463095	69	333205	3466556	41.24	119867	72885	11	20
SFB23	77	40	5443603	5428226	47	990860	5433815	57.22	353835	127287	6	11
